# Left Vocal Cord Paralysis in Idiopathic Pleuroparenchymal Fibroelastosis: A Case Report

**DOI:** 10.31662/jmaj.2022-0196

**Published:** 2023-03-13

**Authors:** Takayuki Takimoto, Hiromitsu Sumikawa, Toru Arai, Yoshikazu Inoue

**Affiliations:** 1 Department of Internal Medicine, National Hospital Organization Kinki-Chuo Chest Medical Center, Sakai, Japan; 2 Department of Radiology, National Hospital Organization Kinki-Chuo Chest Medical Center, Sakai, Japan; 3 Clinical Research Center, National Hospital Organization Kinki-Chuo Chest Medical Center, Sakai, Japan

**Keywords:** Pleuroparenchymal fibroelastosis, Vocal cord paralysis, Recurrent laryngeal nerve, Pneumonia, Dysphagia

## Abstract

Pleuroparenchymal fibroelastosis (PPFE) is a rare interstitial lung disease that is characterized by predominant upper lobe fibrosis and pleural thickening. In this report, we present an unusual case of idiopathic PPFE with left vocal cord paralysis that developed repeated aspiration pneumonia. Vocal cord paralysis is a rare complication of PPFE, and two mechanisms can be proposed: 1) Fibrous adhesion of the recurrent laryngeal nerve to the chest wall can cause stretching of the nerve. 2) Traction or compression of the recurrent laryngeal nerve due to the distortion of the tracheobronchial tree can cause paralysis of the vocal cord. Finally, to reduce the risk of aspiration pneumonia, laryngoscopic evaluation of the vocal cord is recommended in patients with PPFE with hoarseness and dysphagia for early intervention.

## Introduction

Pleuroparenchymal fibroelastosis (PPFE) is a rare interstitial lung disease that is characterized by predominant upper lobe fibrosis and pleural thickening ^(1), (2), (3)^. It is largely idiopathic, but potential disease-associated factors include stem cell transplant, autoimmune disease, and chemotherapy. Idiopathic PPFE (IPPFE) was included as a group of rare idiopathic interstitial cases of pneumonia in the official American Thoracic Society/European Respiratory Society 2013 classification ^(4)^. Although pneumothorax and pneumomediastinum are frequent, vocal cord paralysis or paresis is a rare complication of PPFE ^(5), (6), (7)^. In this report, we present an unusual case of IPPFE with left vocal cord paralysis that developed repeated pneumonia.

## Case Report

**Figure 1.**
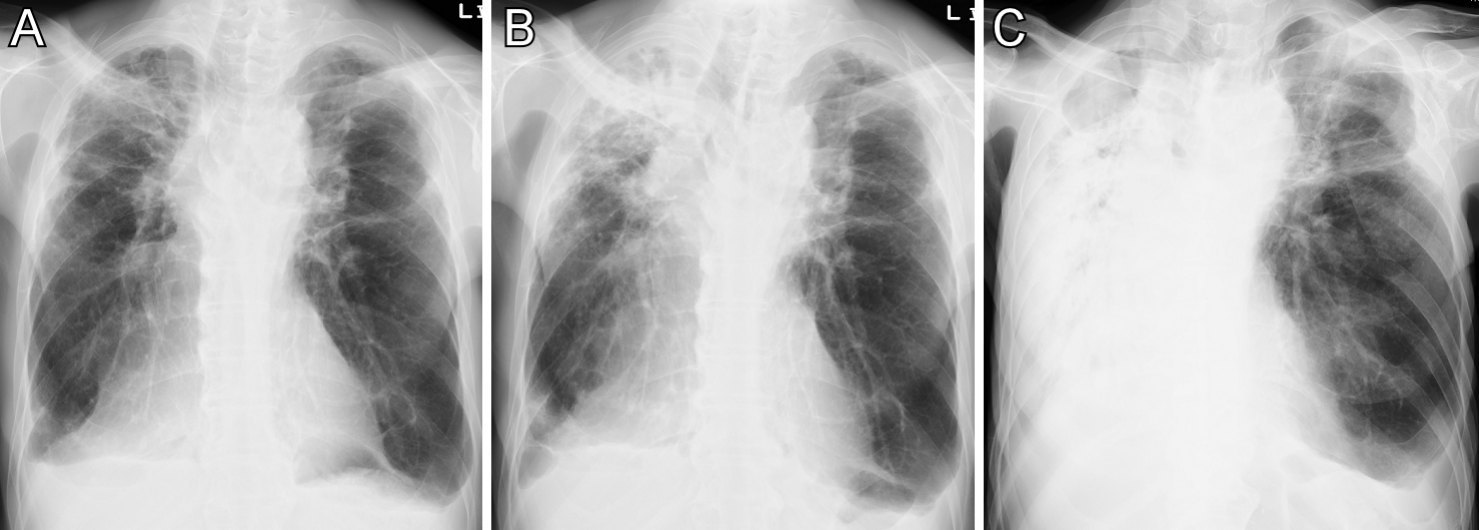
Chest X-ray on initial consultation (A), on admission for pneumonia in the right upper lobe a year later (B), and at the terminal stage with right-sided massive pneumonia a year and three months after the initial consultation (C).

**Figure 2.**
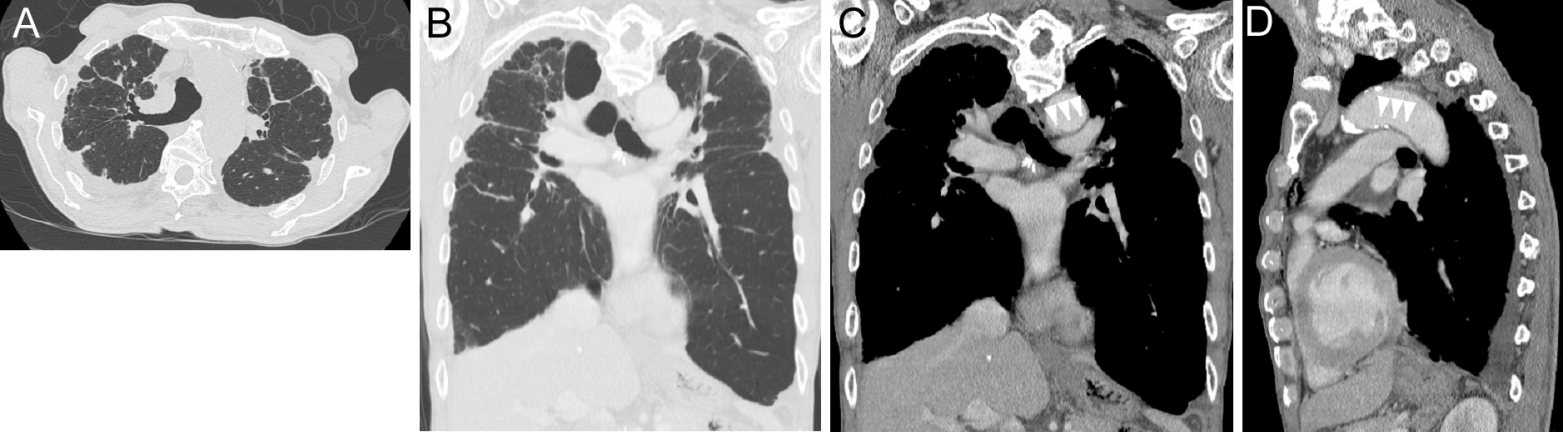
CT of the chest, showing bilateral subpleural predominant consolidation with traction bronchiectasis and volume loss. The lesion was predominant in the right lung, and cysts and traction bronchiectasis were widely observed in the right upper lobe. The volume loss of the right upper lobe was remarkable, and a rightward mediastinal shift was noted (A, transverse view of high-resolution CT on initial consultation; B, coronal view responding to C at the onset of hoarseness 2 months after the initial consultation). The aortic arch and left pulmonary artery were closely contacted, possibly compressing the left recurrent laryngeal nerve (white arrowheads in C, coronal view, and in D, sagittal view at the onset of hoarseness).

A 69-year-old man presented to our hospital with exertional dyspnea. His height was 167.5 cm, and his body weight was 49.9 kg, indicating a body mass index of 17.8 kg/m^2^. X-ray (Figure 1A) and computed tomography (CT) of the chest (Figure 2A) demonstrated bilateral upper lobe fibrotic lesions and volume loss predominant in the right lung, and a rightward mediastinal shift was noted. Laboratory data showed elevated serum levels of Krebs von den Lungen-6 (853 U/ml) and surfactant protein-D (296.9 ng/ml). Autoimmune, hypersensitivity, and infectious serum workups were negative except for a positive anti-SSA autoantibody, and autoimmune diseases were denied by a rheumatologist. Pulmonary function tests showed a restrictive pattern (vital capacity of 59.5% predicted) with a reduced diffusion capacity of 58.9% predicted. Residual volume/total lung capacity was elevated up to 147.5% predicted. Transbronchial biopsy showed peribronchial or airway-centered fibroelastosis, and he was diagnosed with idiopathic pleuroparenchymal fibroelastosis (IPPFE) at a multidisciplinary interstitial lung disease conference. He was treated with no medication. Two months later, he developed progressive hoarseness, dysphonia, and dysphagia and was diagnosed with left-sided vocal cord paralysis by an otolaryngologist. CT of the neck and chest, and magnetic resonance imaging of the brain revealed no underlying cause of the vocal cord paralysis, except for IPPFE. A year after the initial consultation, he was admitted for pneumonia in the right upper lobe (Figure 1B). He had lost 4 kg in weight over a year. A videofluoroscopic examination of swallowing showed delayed swallowing reflex and pharyngeal residue. Fiberoptic laryngoscopy revealed a left-sided mobility disorder of the vocal cord with additional rotation of the left arytenoid cartilage. The closure of the glottis was incomplete (Supplemental Video). Three months later, he died of recurrent pneumonia (chest X-ray in Figure 1C), despite the prevention of aspiration pneumonia by a comprehensive multidisciplinary team approach and treatment with antibiotics.

## Discussion

Vocal cord paralysis or paresis has been rarely reported in PPFE^(5), (6), (7)^, and its underlying mechanism remains unknown. Several mechanisms of vocal cord paralysis have been previously discussed in lung parenchymal diseases ^(8)^. A possible mechanism is proposed, that is, fibrous adhesion of the recurrent laryngeal nerve to the chest wall due to PPFE can cause stretching of the nerve ^(5)^. Generally, unilateral vocal cord dysfunction secondary to mediastinal abnormalities occurs more occasionally on the left due to the course and length of the left recurrent laryngeal nerve ^(8), (9)^. There have been three case reports, except for our case, that describe vocal cord paralysis or paresis in PPFE ^(5), (6), (7)^. Three out of the four cases, including our case, were left-sided. Another possible mechanism is that the traction or compression of the recurrent laryngeal nerve due to the distortion of the tracheobronchial tree can cause paralysis of the vocal cord ^(5), (8), (10), (11)^. In this case, CT revealed that the aortic arch and left pulmonary artery were closely contacted due to the distortion of the tracheobronchial tree toward the right side (Figure 2B, C, D), possibly suggesting the compression of the left recurrent laryngeal nerve, as in cases with pulmonary artery enlargement or aortic arch aneurysm ^(8), (11)^. In this case, vocal cord paralysis was probably not affected by bronchoscopy, because it did not deteriorate just after the bronchoscopy but deteriorated after a while within 2 months.

Prevention of aspiration pneumonia and pulmonary exercise training should be important for maintaining respiratory function in patients with IPPFE. In the earlier stage of this case, oral ingestion could be avoided using a tracheostomy with a blocked tracheostomy tube to prevent aspiration pneumonia, and supplemental intestinal nutrients are considered to enhance the nutritional state. To reduce the risk of aspiration pneumonia, laryngoscopic evaluation of the vocal cord is recommended in patients with IPPFE with hoarseness and dysphagia for early intervention.

## Article Information

### Conflicts of Interest

TT has received lecture fees from Shionogi & Co., Ltd.

TA has received lecture fees from Shionogi and Boehringer Ingelheim for activities not connected with this work.

YI is a consultant or steering/advisory committee member for Boehringer Ingelheim, Roche, SAVARA, and Taiho. Yoshikazu Inoue has received lecture fees from Boehringer Ingelheim, 20 Shionogi, Kyorin, Thermo Fisher, and GSK. However, all of them were not related to the current report.

### Author Contributions

T.T. managed the patient. H.S., T.A., and Y.I. assisted preparation of the manuscript. All authors had access to the data and played a role in writing the manuscript. Written consent for publication was obtained.

### Informed Consent

The patient provided written informed consent to publish this case report and accompanying images.

## Supplement

Supplemental Video Fiberoptic laryngoscopy, showing the paralysis of the left vocal cord, besides atrophy and poor movement of the bilateral vocal cord.
